# Newborn literacy program effective in increasing maternal engagement in literacy activities: an observational cohort study

**DOI:** 10.1186/1471-2431-12-100

**Published:** 2012-07-16

**Authors:** Stephanie Veldhuijzen van Zanten, Chrystal Coates, Marilou Hervas-Malo, Patrick J McGrath

**Affiliations:** 1 Dalhousie University, Halifax, NS, Canada; 2 SABA University School of Medicine, Saba, Netherlands; 3 Epicore Centre, Faculty of Medicine, University of Alberta, Edmonton, AB, Canada; 4 IWK Health Centre, Canada Research Chair, Professor of Psychology, Pediatrics and Psychiatry, Dalhousie University, Halifax, NS, Canada

## Abstract

**Background:**

Literacy is important for success in school and in adulthood. Book-gift programs at birth exist to help develop these foundations early on. The effectiveness of the Read to Me! Nova Scotia Family Literacy Program (a program where books and literacy materials are given to families in hospital when their baby is born) on the duration and frequency with which mothers engage in reading and other literacy based activities with their newborns was assessed.

**Methods:**

An observational cohort study design was used. Mothers of babies who received the Read to Me! package in Nova Scotia born between January-August 2006 made up the intervention group (N = 1051). Mothers of babies born in Prince Edward Island between December 2006 and March 2008 made up the control group (N = 279) and did not receive any literacy package when their baby was born. A phone questionnaire was conducted consisting of questions regarding frequency and duration of maternal engagement in language and literacy-based activities with their infants. These activities included reading, singing, talking, listening to CDs and the radio and watching TV. Babies were aged 0–10 months at the time of the interview.

**Results:**

Mothers who received the Read to Me! literacy package spent significantly more time reading to their babies, 17.9 ± 17.6 min/day compared to controls 12.6 ± 10.7 min/day, (p < 0.0001).

**Conclusions:**

Read to Me! may be an inexpensive, easy to administer and effective intervention which results in increased shared reading of mothers and their newborns.

## Background

Reading is an important component of literacy but does not begin until just prior to or after school entry. The foundational skills that precede reading are developed before entering elementary school [1] and parents can play a pivotal role in developing these skills with their children. Children who experience difficulty with reading and literacy early on often will continue to suffer from low literacy and have problems that persist into the school years [2].

Several studies have found a positive relation between storybook exposure and vocabulary in kindergarten [3-5]*.* Frequency of storybook reading is not the only important factor in the development of literacy skills in young children, but it can influence vocabulary and other language and reading behaviours. This relationship has primarily been explored in toddlers and children in early elementary school. There has been limited exploration of this in children from birth through infancy. The finding that maternal attitudes toward shared reading in infancy and resources (e.g. number of baby books in the home) are predictors of parental engagement and reading behaviours at age 6 months [6] points to the need to evaluate ways of positively influencing these behaviours in mothers and families from birth. This can include anticipatory guidance such as education as to the benefits of early reading and/or book-gift programs.

Many early literacy intervention programs exist including Reach Out and Read in the United States, Bookstart in the United Kingdom and Read to Me! in Nova Scotia, Canada. The Read to Me! program gives a gift of books and other literacy materials to parents at the hospital bedside prior to leaving hospital after the birth of a baby.

The purpose of the current study is to examine the effectiveness of the Read to Me! program in Nova Scotia (NS) on the frequency with which mothers engage in literacy activities with their infants compared to mothers of newborns in Prince Edward Island (PEI) who did not receive a literacy intervention. It was hypothesized that mothers who receive the Read to Me! program will engage in reading and other active literacy based activities, such as singing and talking, with their newborn more often and for longer periods of time than those mothers who received no intervention. Engagement in passive activities such as watching TV, listening to CDs and listening to the radio were also compared between groups.

## Methods

### Study design

This study was an observational cohort study comparing the frequency and duration of maternal engagement in reading and other literacy activities with their infants. Mothers who received the Read to Me! Nova Scotia Family Literacy program (intervention group) were compared to mothers in Prince Edward Island who received no newborn literacy package (control group).

### Study sample

#### Intervention group

Mothers in the intervention group were recipients of a literacy package provided by the Read to Me! program. This is a provincial program in Nova Scotia where every family receives a literacy package at the hospital bedside within 24–48 hours of the birth of their baby. This package contains age-appropriate books, a music CD, a booklet containing information on literacy activities and resources, an educational DVD, a library guide and coupons to local bookstores. At the time of the questionnaire the Read to Me! package was available in English and French.

Mothers of babies born between January and August 2006 at the IWK Health Centre in Halifax, NS were asked for their consent to be contacted for the study. Inclusion criteria were that parents had to be able to communicate in English. Infants who were older than 47 weeks (10 months) were excluded as too few parents of infants greater than 10 months could be contacted.

A total of 1200 parents were contacted by phone within 10 months of the birth of their baby and asked to complete a 10–15 minute phone questionnaire. Of these, 1072 completed the questionnaire and the remaining 128 declined. Reasons for declining were not recorded. The majority of participants were the mothers of the babies (mothers: 99%, n = 1051, fathers: 0.8%, n = 9, other relative: 0.1%, n = 1) of the babies. In order to maintain a uniform sample, the 10 participants who completed the study who were not the mother of child were excluded. 4 participants were excluded because their babies were too old. The remaining 1051 mothers made up the intervention group for the study.

#### Control group

The control group for this study was made up of parents who had children born between December 2006 and March 2008 at the Queen Elizabeth Hospital in Charlottetown, PEI. All parents who had babies within this time period received a mail-out within a few months of the birth of their baby. This included a letter signed by pediatricians in PEI encouraging participation in the study as well as a letter from the principal researcher outlining the study and asking them to participate. Parents were given an email contact and toll-free phone number to call if they were interested in participating in the study. Follow-up phone calls were also conducted by a local ward clerk to increase recruitment. Prior to November 2006, a newborn book-gift program was in place in PEI but this was stopped due to lack of funding.

Of the 1095 letters that were mailed out, 133 parents responded by phone or email and an additional 219 were recruited by the ward clerk. A total of 352 parents of babies aged 0–9 months born in Prince Edward Island (PEI) were contacted to participate. Of these 300 completed the phone questionnaire. 18 participants were excluded because they reported receiving a literacy package at the time their baby was born and 1 was excluded because the child was too old at the time the phone questionnaire was conducted. To maintain a uniform sample 2 participants were excluded because a family member other than the mother completed the questionnaire. The remaining 279 mothers made up the control group for the study.

**Figure 1:**
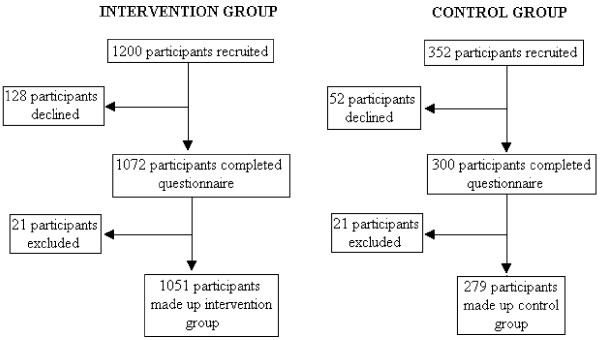
Selection of intervention and control groups.

Selection of intervention and control groups is shown in Figure 1.

### Study questionnaire

The phone questionnaire was conducted by trained interviewers. Verbal consent over the phone was obtained when parents were contacted about their interest in participating. The questionnaire consisted of questions about language and literacy-based activities in which parents engage with their babies. These activities were measured in terms of frequency, as number of times per week and duration, number of minutes per day. These activities included singing to their baby, playing songs on CD, reading, listening to the radio, watching television or talking directly to their baby.

Demographic questions were also asked including number of adults and children in the home, age, occupation and highest level of education of parents as well as total income for the home. This information was used to determine if these factors are predictors of how often parents read to their babies.

The phone questionnaire was initially created by the authors to evaluate the Read to Me! Program and its specific components. The questionnaire has not been validated. With the addition of the control group the same phone questionnaire, with the exception of questions about the Read to Me! components, was used. This allowed for direct comparison between the two groups. All responses were entered into an electronic database.

Research ethics approval for the study was obtained from both the IWK Health Centre, NS and the Queen Elizabeth Hospital, PEI.

### Statistical analysis

The frequency and duration of mothers’ engagement in literacy activities with their infant was assessed by measuring the amount of time spent participating in these activities in the week preceding the phone call. Mothers were also asked how many minutes each day they engaged in these activities as well as how many days per week. Mothers who reported they did not participate in a given activity or those who reported not having engaged in the activity in the last week were coded as zeroes for the purposes of analysis. Participants who indicated they participated in a particular activity but did not specify the total time spent on the activity were recorded as missing. As a result there was a different set of sample size for yes/no activity and duration of time spent doing activity outcomes.

A descriptive summary consisting of frequencies and percentages for categorical variables, means with standard deviation and medians with inter-quartile ranges, as well as minimum and maximum for continuous variables were tabulated for all questions collected in order to assess the differences if any between the intervention and control groups. p values were calculated using chi-square tests, t-tests or Mann Whitney test as appropriate for the primary outcome measures. Since the time to engaging in an activity can in fact be interpreted as duration or the number of times doing each activity, the response variable was then treated as a Poisson random variable but to accommodate over-dispersion the parametric model Negative Binomial was assessed and showed better fit. P values of < 0.05 were considered significant. Unadjusted and adjusted odds ratios and p-values using logistic and risk ratios using negative binomial models were examined. SAS 9.1 (Cary, N.C.) was used in all statistical analysis.

In the multivariate analysis, the variables used were group, age of primary parent, income level, education level and number of the adults in the home.

## Results

### Sample characteristics

The group comparisons of parent and baby characteristics showed no marked difference between the intervention and control group. The demographic characteristics are shown in Table 1. There were no significant differences in income, maternal education level and age of the mother among the two groups. There was no overall difference in age of babies between the two groups, however a sub-analysis did result in a higher number of infants aged 4–6 months.

**Table 1:** Descriptive data for intervention and control groups

	**Intervention (n = 1051)**	**Control (n = 279)**	**p value**
	**Nova Scotia**	**PEI**	
Age of primary parent (mean ± SD)	N = 1051, 31 ± 5	N = 278, 32 ± 5	0.3
Income Level			
Low (<60 K)	261 (24.8%)	74 (26.5%)	0.35
Medium (60–99)	314 (30.0%)	88 (31.5%)	
High (100 & up)	201 (19.1%)	54 (19.4%)	
Did Not Answer	270 (25.7%)	63 (22.6%)	
Missing	5 (0.5%)		
Education Level			
Less than/HS	201 (19.1%)	54 (19.4%)	0.93
College/Univ/Grad	850 (80.9%)	225 (80.6%)	
Number of adults in home			
One	35 (3.3%)	7 (2.5%)	0.68
Two or more	1015 (96.6%)	272 (97.5%)	
Missing	1 (0.10%)	0	
Baby’s age in months (mean ± SD)	5.6 ± 2.9	5.4 ± 2.0	0.07

#### Outcomes

Mothers who were recipients of the Read to Me! literacy program spent significantly more time reading to their babies, 17.9 ± 17.6 min/day compared to controls 12.6 ± 10.7 min/day, (p < 0.0001). After adjustment for all other predictor factors in the model, the conclusion remained the same with mothers in the intervention group reading 1.4 times more (RR: 1.4 95% CI: (1.2-1.6)) than mothers in the control group. Mothers in the intervention group also engaged in singing to their baby more than mothers in the control group, 47.2 ± 43.3 min/day as compared to 41.4 ± 36.5 min/day (p = 0.04). The strength of the association however did not persist after adjustment for other factors in the model.

The number and proportion of parents in the control group who watched TV with their children were significantly higher than those of parents in the intervention group (68.5% vs 59.8%). However, there was no statistical difference between groups in the amount of time babies watched TV. Data for engagement in both active and passive activities are presented in Table 2 and Table 3.

**Table 2:** Proportion of parents and average time spent engaging in active and passive activities

	**Intervention**	**Control**	**Chi-square P value**	**Unadjusted Odds Ratio (OR)**	**Adjusted Odds Ratio (OR)**
	**(n = 1051), n (%)**	**(n = 279), n(%)**		**(95% CI)**	**(95% CI), P value**
**Active Activities**					
Read	957 (91.1)	245 (87.8)	0.10	1.4 (0.9-2.1)	1.5 (0.9-2.4), 0.13
Sing	1028 (97.8)	273 (97.8)	0.97	1.1 (0.4-2.4)	.2 (0.4-3.2), 0.78
Talk	1046 (99.5)	279 (100)	0.25	Not estimable	Not estimable
**Passive Activities**					
Listen to CDs	665 (63.3)	169 (60.6)	0.41	1.1 (0.9-1.5)	1.2 (0.8-1.6), 0.37
Watch TV	629 (59.8)	191 (68.5)	0.01	0.7 (0.5-0.9)	0.6 (0.5-0.9), 0.01
Listen to radio	667 (63.6)	190 (68.1)	0.16	0.8 (0.6-1.1)	0.8 (0.5-1.0), 0.08

**Table 3:** Participant outcomes, daily average time spent in each activity (in minutes/day)

	**Intervention**		**Control**		**Chi-square P value**	**Unadjusted Risk Ratio (RR)**	**Adjusted Risk Ratio (RR)**
	**N**	**Mean (SD)**	**n**	**Mean (SD)**		**(95% CI)**	**(95% CI), p value**
**Active Activities**							
Read	1051	17.9 ± 17.6	279	12.6 ± 10.7	<0.0001	1.4 (1.2-1.6)	1.4 (1.2-1.6), <0.0001
Sing	1048	47.2 ± 43.3	279	41.4 ± 36.5	0.04	1.1 (1.0-1.3)	1.1 (0.9-1.2), 0.24
Talk	1034	238.8 ± 160.75	279	232.7 ± 134.3	0.61	1.0 (0.9-1.1)	1.0 (0.9-1.1), 0.94
**Passive Activities**							
Listen to CDs	1045	20.1 ± 36.3	279	16.4 ± 23.3	0.14	1.2 (0.9-1.6)	1.2 (0.9-1.7), 0.19
Watch TV	1050	18.1 ± 25.5	279	22.3 ± 28.9	0.11	0.8 (0.6-1.05)	0.8 (0.6-1.0), 0.05
Listen to Radio	1048	44.9 ± 73.8	279	49.0 ± 76.8	0.53	0.9 (0.7-1.2)	0.9 (0.6-1.2), 0.4

Predictors of reading and listening to CDs are shown in Table 4. Age of the baby was a significant predictor in all of the activities, showing that for each monthly increase in the age of the baby, the likelihood of parents engaging in each activity increased by roughly 8-40% (p < 0.001) after adjustment. Parents who finished post secondary education compared to those with high school or lower education were more likely to read to their babies. Interestingly, parents with medium to high income were more likely to engage in listening to CDs compared with parents with lower income.

**Table 4:** Modeling predictors of reading and listening to CDs using logistic regression

	**Reading**		**Listening to CDs**	
	**OR (95% CI)**	**pvalue**	**OR (95% CI)**	**p value**
**Group**				
Intervention Group	1.5 (0.9-2.4)	0.13	1.2 (0.8-1.6)	0.37
Control Group	1.0		1.0	
**Age of primary parent**				
Continuous variable	0.96 (0.91-1.01)	0.09	0.98 (0.96-10.1)	0.34
**Income Level**				
Low (<60 K)	1.0		1.0	
Medium (60–99)	1.0 (0.6-1.7)	1.0	1.3 (1.0-1.8)	0.08
High (100 & up)	0.8 (0.4-1.6)	0.6	2.1 (1.4-3.1)	0.0001
**Education Level**				
Less than/HS	1.0		1.0	
College/Univ/Grad	2.7 (1.4-5.0)	0.002	1.3 (0.8-1.9)	0.25
**Number of adults in home**				
One	1.0		1.0	
Two or more	0.6 (0.1-3.1)	0.6	0.9 (0.4-2.0)	0.80
**Baby’s age in months**				
Continuous variable	1.3 (1.2-1.4)	<0.0001	1.1 (1.03-1.14)	0.001

For reading, talking and watching TV activities, age of baby was a significant factor. As infants got older, parents increased their time spent in doing these activities with them. The age of the parent was a significant predictor as well in reading and talking activities. That is, younger parents were more likely to read or talk to their babies than older parents.

The results for modeling the time spent reading, singing, talking and watching TV (adjusted models) are shown in Table 5. Age of the parent, education level of parent, baby’s age and having received the Read to Me! intervention were all predictors of increased reading.

**Table 5:** Modeling the average number of minutes per day spent in each active activity and watching TV

	**Reading**		**Singing**		**Talking**		**Watching TV**	
**Predictors**	**RR (95% CI)**	**P value**	**RR (95% CI)**	**P value**	**RR (95% CI)**	**P value**	**RR (95% CI)**	**P value**
**Group**								
Intervention	1.4 (1.2-1.6)	<0.0001	1.09 (0.94-1.25)	0.24	0.99 (0.89-1.1)	0.94	0.8 (0.6-1.0)	0.054
Control			1.0		1.0		1.0	
**Age of primary parent**								
Continuous variable	0.98 (0.97-0.98)	0.02	1.0 (0.99-1.02)	0.67	0.99 (0.98-0.99)	0.03	0.99 (0.97-1.02)	0.51
**Income Level**								
Low (<60 K)	1.0		1.0		1.0		1.0	
Medium (60–99)	1.1 (0.9-1.3)	0.27	1.05 (0.9-1.2)	0.5	0.99 (0.9-1.1)	0.83	0.8 (0.6-1.1)	0.11
High (100 & up)	1.1 (0.9-1.3)	0.33	1.04 (0.9-1.2)	0.67	0.9 (0.8-1.05)	0.22	0.8 (0.6-1.1)	0.23
**Education Level**								
Less than/HS	1.0		1.0		1.0		1.0	
College/Univ/Grad	1.3 (1.1-1.6)	0.01	0.96 (0.8-1.1)	0.65	1.02 (0.9-1.1)	0.82	0.8 (0.6-1.2)	0.36
**Number of adults in home**								
One	1.0		1.0		1.0		1.0	
Two or more	0.8 (0.6-1.2)	0.39	0.9 (0.6-1.4)	0.71	0.95 (0.7-1.3)	0.73	0.8 (0.4-1.8)	0.67
**Baby’s age in months**								
Continuous variable	1.05 (1.02-1.07)	0.0001	1.0 (0.98-1.03)	0.68	1.05 (1.03-1.07)	<0.0001	1.1 s(1.04-1.1)	0.006

## Discussion

In this study mothers who received the Read to Me! literacy package in the hospital when their baby was born read more to their babies compared to mothers who did not receive it. These findings provide evidence that Read to Me! is an effective intervention to increase the amount babies are read to. We believe that the 5 minute increase in reading time is clinically relevant because it represents a substantial increase in the intervention group as compared to the control group. The control group read 12 minutes a day and a 5 minute increase therefore is a 40% increase. Studies have shown that the earlier children are read to the greater the effect on later language skills. If interventions, such as Read to Me! can significantly increase time spent reading then this may have a meaningful impact on later literacy skills. We had not a priori defined what amount of improvement in reading time would be clinically meaningful but are confident that the results support the benefit of the Read to Me! program.

Previous studies have shown that the earlier children start to be read to, the greater the effect on language skills [7,8]. Our result, in the context of such studies, may be clinically meaningful as it suggests that interventions which substantially increase engagement in literacy activities with children starting at birth may positively affect later language and literacy skills.

It was originally intended that infants older than 10 months were to be included but we were unable to contact many infants greater than 10 months so they were excluded. The lower range started from birth as there are few studies that exist which focus on literacy activities in this age group and we felt it was important to examine this further.

It is well established that the foundations for literacy begin well before children are able to read. There is evidence that the development of literacy skills begins at birth, continues through schooling and is related to later academic success [9]. Children’s early experiences including exposure to books, early language and literacy skills, are important contributors to reading ability in grade school. [10]*.*

Using a cumulative risk model Cadima et al. [11] found that both preschool and first grade literacy skills were influenced by family risk factors such as number of parents in the home, income and maternal education. This highlights the importance of creating literacy interventions which target children before they reach preschool. Many studies have been conducted which look at reading and its precursors but few have focused on interventions targeted at infants.

Book-gift programs, such as Reach Out and Read! in the United States, have been well studied and have proven to be effective interventions for encouraging shared reading in the home for preschool-aged children [12-15] but evidence for children under 1 year is lacking.

The attitudes of mothers with regards to shared reading and resources available to them (i.e. number of baby books in the home) at the birth of their baby are both predictors of shared reading at 6 months [6]. This would suggest that providing additional resources to mothers at the birth of their baby, such as with a package similar to Read to Me!, may influence their attitudes and in turn may encourage engagement in shared reading beginning in early infancy. The present study has shown this simple intervention to be an effective means of increasing shared reading between mothers and their newborns. By providing age-appropriate baby books as well as other resources to parents while in the hospital, Read to Me! emphasizes the importance of engaging in shared reading right from birth.

The findings of this study are important because it is one of the few to study literacy interventions targeted at newborns and show that they are successful. Read to Me! has many aspects which make it unique from other book-gift programs aside from the age-group it targets. Read to Me! is given at the hospital bedside which is a near universal delivery point and allows this program to be delivered to virtually every child born in the hospital. Read to Me! is also an inexpensive and easily delivered intervention. The cost of a single bag which contains 2 children’s books, a book with nursery rhymes, a CD with nursery songs, information about libraries and other literacy resources, and the bag itself is $27.19 CDN. The Read to Me! bag is now also available in Mi’ kmaq and Arabic in addition to English and French.

Further research needs to be conducted to evaluate the types of interactions parents are having with their infants during shared book reading as well as a comparison of book behaviours in children who did receive an intervention compared to those who did not. A longitudinal evaluation of children who received book-gift bags as infants should be conducted to determine if these interventions have an impact on fostering long-term positive associations with reading and on later language and literacy abilities.

This study has several strengths. Firstly, the sample size for both the intervention and control groups was large. It was not possible to have equal numbers in both groups due to the difference in population size of the two geographic areas from which the participants were recruited. Secondly a wide variety of language and literacy based activities were included and both duration and frequency with which mothers engaged in both active and passive activities was explored.

The study did have several limitations, including the fact that it was a non-randomized study which was dependent on self-report. The proportion of babies aged 4–6 months was slightly higher in the control group. It would be expected that if anything this difference in age would make the differences between the control and intervention groups smaller because older age was a predictor of more frequent engagement in literacy activities. Given the differences in recruitment between the intervention and control groups, there may have been selection bias present in the control group because recruitment was via mail requests with a follow-up phone call from the ward clerk. However, given the large sample size of the interventions group, which was recruited prior to the control group, it was felt that the recruitment method was appropriate to increase the sample size of the control group as much as possible.

Numerous other variables such as the number of siblings in the home, the style of reading and age-appropriateness of books may influence the mother’s engagement in literacy activities and effects of the engagement. These should be explored in future studies but were beyond the scope of this paper.

## Conclusions

In summary, we have demonstrated that Read to Me! may be an inexpensive, easy to administer and effective intervention resulting in increased shared reading of mothers and their newborns.

## Competing interests

The authors declare that they have no competing interests.

## Authors’ contributions

SV participated in the study design, data collection and drafted the manuscript. CC participated in the study design, data collection and analysis and helped to draft the manuscript. MH performed the statistical analysis and interpretation of data. PM conceived of the study, participated in its design and coordination and helped to draft the manuscript. All authors read and approved the final manuscript.

## Pre-publication history

The pre-publication history for this paper can be accessed here:

http://www.biomedcentral.com/1471-2431/12/100/prepub
